# Drivers and constraints on offshore foraging in harbour seals

**DOI:** 10.1038/s41598-021-85376-2

**Published:** 2021-03-22

**Authors:** H. M. Vance, S. K. Hooker, L. Mikkelsen, A. van Neer, J. Teilmann, U. Siebert, M. Johnson

**Affiliations:** 1 SMRU (Sea Mammal Research Unit), University of St Andrews, St Andrews, Fife, KY16 8LB UK; 2 Institute for Terrestrial and Aquatic Wildlife Research, University of Veterinary Medicine Hannover, Foundation, Werftstraße 6, 25761 Büsum, Germany; 3 Marine Mammal Research, Department of Bioscience, Aarhus University, Roskilde, Denmark; 4 Aarhus Institute of Advanced Studies and Department of Biology, Aarhus University, Aarhus C, Denmark

**Keywords:** Behavioural ecology, Animal behaviour

## Abstract

Central place foragers are expected to offset travel costs between a central place and foraging areas by targeting productive feeding zones. Harbour seals (*Phoca vitulina*) make multi-day foraging trips away from coastal haul-out sites presumably to target rich food resources, but periodic track points from telemetry tags may be insufficient to infer reliably where, and how often, foraging takes place. To study foraging behaviour during offshore trips, and assess what factors limit trip duration, we equipped harbour seals in the German Wadden Sea with high-resolution multi-sensor bio-logging tags, recording 12 offshore trips from 8 seals. Using acceleration transients as a proxy for prey capture attempts, we found that foraging rates during travel to and from offshore sites were comparable to offshore rates. Offshore foraging trips may, therefore, reflect avoidance of intra-specific competition rather than presence of offshore foraging hotspots. Time spent resting increased by approx. 37 min/day during trips suggesting that a resting deficit rather than patch depletion may influence trip length. Foraging rates were only weakly correlated with surface movement patterns highlighting the value of integrating multi-sensor data from on-animal bio-logging tags (GPS, depth, accelerometers and magnetometers) to infer behaviour and habitat use.

## Introduction

Central place foragers must access food resources that compensate the cost of travel between feeding areas and the central place. As travel distance increases, extra energy must be obtained by either foraging for longer or by targeting sites with denser or richer prey^1^. The need to access rewarding food resources can drive aquatic central place foragers, such as pinnipeds, to make lengthy trips away from coastal haul-out sites. Ross seals (*Ommatophoca rossii*) and southern elephant seals (*Mirounga leonina*) famously travel 1000’s of km in multi-month foraging trips^2,3^. Antarctic fur seals (*Arctocephalus gazella*) that travel to more distant patches have more high-energy prey in their diet than those foraging closer to their haul-out sites^4^. Likewise, distance travelled during foraging trips is positively correlated with estimated mass gain in male northern elephant seals (*Mirounga angustirostris*), indicating that travel costs are more than recouped by energetic returns^5^.

Harbour seals (*Phoca vitulina*) are primarily a coastal species but also travel offshore for days at a time, often journeying > 100 kms from haul-out sites^6–8^. However, some individuals target resources closer to the haul-out^6,8,9^, raising the question: why do some individuals accept the extra travel costs associated with foraging further offshore? Indeed, it has been suggested that some populations, such as the Wadden Sea harbour seals, feed almost exclusively at offshore sites suggesting that these sites provide a resource which is not available closer to the haul-out^6,8,10,11^.

The Wadden Sea World Heritage Area (henceforth Wadden Sea), located off the north-west coasts of the Netherlands, Germany and south-west Denmark, extends some 12 nautical miles into the North Sea. It is characterised by a flat, mainly sandy seabed reaching depths of 10–20 m^12^ and is home to a regionally important population of harbour seals^13^. This shallow sea is fringed by tidal mudflats and a highly productive estuarine habitat^14^. Although the apparent homogeneity of this environment seems at odds with the idea of rich offshore prey patches that could attract harbour seals away from the coast and into the North Sea, fisheries research suggests that the distribution of resources here may not be uniform. Coastal waters of the Wadden Sea are important nursery grounds for prey species such as flatfish and sand eel^15,16^, with larger individuals moving offshore as they mature. There has also been a shift in the distribution of fish assemblages into deeper water with increasing sea temperatures^17^, suggesting that more and larger prey may be available offshore. Increased predation close to haul-out sites may also reduce prey abundance around coastal sites. Such zones of depletion, known as Ashmole's halo^18^, have been observed around seabird colonies and drive predators further offshore to forage.

Optimal foraging models predict that animals travelling further should forage longer to recoup travel costs, but trip length is likely constrained by other factors e.g., the need for sleep, digestion and/or social interaction. Indeed, the haul-out must provide important resources for harbour seals for them to give up valuable foraging opportunities having invested the costs of traveling offshore. However, as harbour seals are able to both rest and digest whilst at sea^19–22^, it is unclear what factors limit the duration of these trips outside of the breeding and moulting season. Thus, despite the ubiquity of harbour seals around populated northern European coastlines, relatively little is known about what drives and constrains their offshore foraging.

Harbour seal behaviour has generally been studied with telemetry tags that transmit low-resolution dive depth and position data over periods of months to years^23^. To infer intervals of foraging from these data, it is assumed either that animals produce more tortuous surface tracks when foraging (i.e., by turning to remain within a prey patch^24^, or that their dive profiles correspond to shapes which are suggestive of foraging e.g., U-shaped dives imply more time invested in a presumed prey layer located at the base of the dive as compared to V-shaped dives which may represent travel^25^. However, both metrics have limitations for classifying behaviours: U-shaped dive profiles are obtained if an animal remains stationary on the bottom, such as during resting^21,22^ and so can be confused with foraging when using low resolution data. Likewise, infrequent location data risks under-sampling tortuosity thereby placing a lower limit on the patch size that can be detected. For these and other reasons, e.g., inaccuracy of location data and insufficient validation of foraging indicators (for a review see Carter et al.^23^), inferences based on low resolution data may not provide reliable information on activity budgets and foraging rates. For harbour seals, behavioural categorisation based on telemetry data has received sparse independent validation making it difficult to assess reliability. Dive shape behavioural categorisation, validated via stomach temperature telemetry^26^ and video analysis^27^, has given mixed results. Lesage, Hammill and Kovacs^26^ suggest that all dive shapes are employed during foraging while Baechler et al.^27^ found that dive shape was less reliable as a foraging indicator for males in the breeding season. Behavioural inferences from surface tracks have yet to be validated for harbour seals to the best of our knowledge. For another phocid, the southern elephant seal, several studies have shown tortuosity of surface tracks to be a good predictor of foraging behaviour^28,29^, but one study reported extensive foraging during both directed travel and intensive search modes^30^.

In recent years, archival multi-sensor bio-logging tags have been used increasingly to investigate fine-scale behaviour of pinnipeds^22^. Low-power movement sensors, such as accelerometers and magnetometers can track the orientation of animals as well as faster actions such as swim strokes and prey strikes^31–35^. Accelerometers have been employed on a number of pinniped species to detect prey capture attempts^33,36,37^ and sharp changes in acceleration appear to be reliable indicators of both suction and raptorial feeding in harbour seals^37^, making this a useful sensor to quantify foraging rates. Combining data from multiple sensors can provide a more definitive picture of an animal’s fine-scale movements and the context within which they are performed^8,38–40^.

Here we use multi-sensor bio-logging tags, incorporating GPS, depth sensors, accelerometers and magnetometers to study offshore foraging in harbour seals of the Wadden Sea at a fine temporal and spatial scale. Specifically we test the hypotheses that: (1) animals travel offshore to access rich foraging patches not available closer to shore, (2) the need for rest influences the decision to return to the haul-out, and (3) low-resolution location and dive data provide reliable inferences of foraging behaviour in harbour seals.

## Results

**Figure 1:**
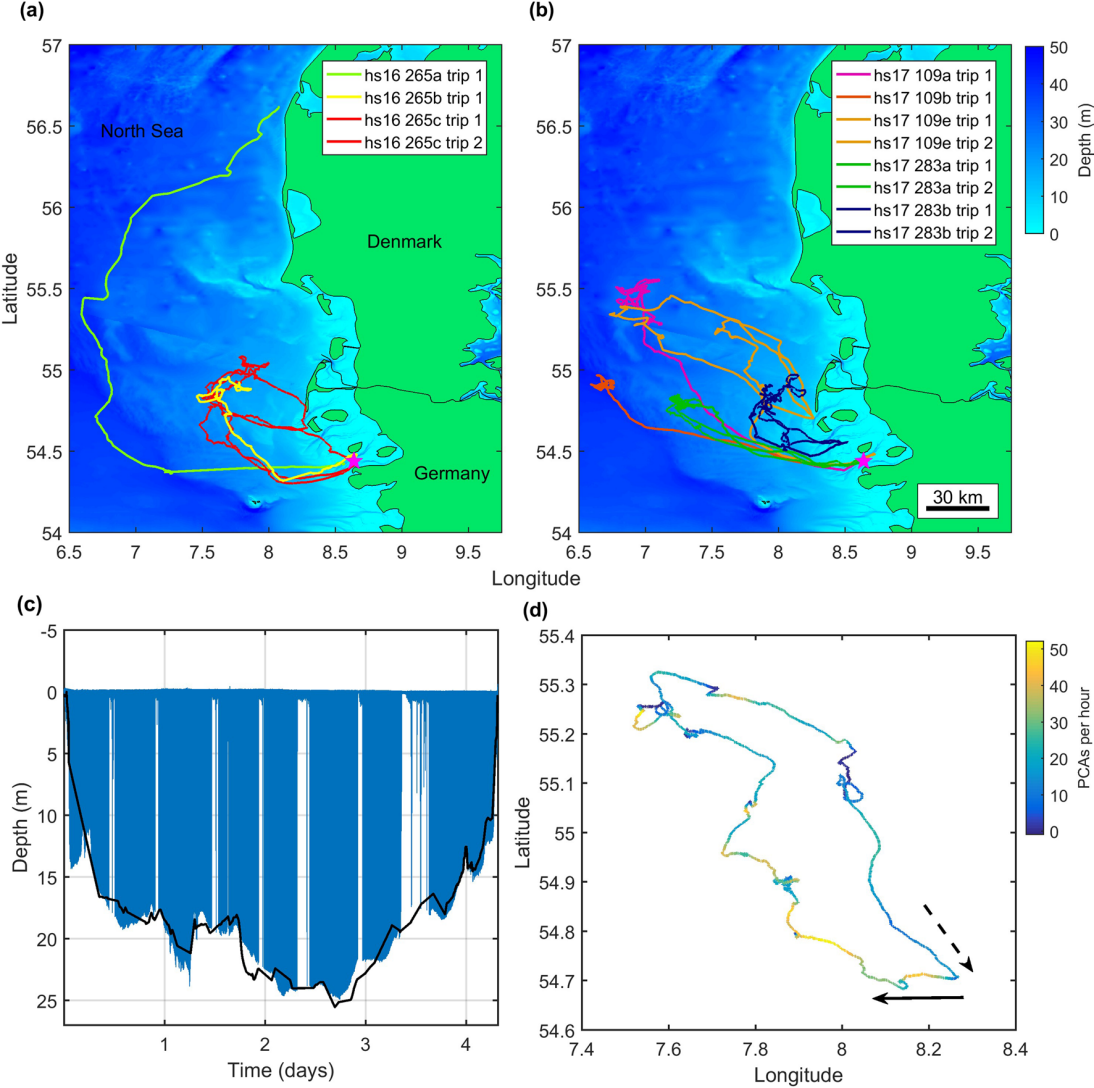
(**a**) GPS surface tracks of animals tagged in 2016. These represent 4 multi-day foraging trips by 3 individuals. One seal (hs16_265a) did not return to the same haul-out site but rather travelled north to Limfjord in Denmark. (**b**) GPS surface tracks of animals tagged in 2017. These represent 8 multi-day foraging trips performed by 5 animals. In six of these offshore trips (2016 and 2017), the tags stopped recording before the seal returned to shore. The tagging site is represented in both (**a**) and (**b**) by a purple star. (**c**) Dive profile of hs17_109e trip2 with the black line indicating bathymetry interpolated from www.emodnet.eu/bathymetry. (**d**) Dead-reckoned horizontal track of hs17_109 trip 2, coloured by the hourly rate of presumed prey capture attempts (PCAs), inferred from acceleration, showing continuous foraging during both straight-line travel to and from offshore prey patches and during more tortuous movements while offshore. The continuous arrow indicates the track of the animal moving away from the haul-out towards offshore areas, while the broken arrow indicates the track of the animal returning to the haul-out.

Of the ten seals tagged, eight (three in 2016 and five in 2017) made 12 offshore foraging trips during 6–30 days of individual tracking (Fig. 1a,b, Table 1). The two additional seals (both in 2017) remained inshore performing only short foraging trips (< 1 day) and were thus excluded from analysis. In half of the offshore trips, tag recordings ended before the animal returned to the haul-out. Therefore, these were only analysed in part. Of the 6 complete foraging trips, from five individuals, the mean trip length was 7 days (SD 2.3 days). Over all trips, seals performed a mean of 13 foraging dives per hour (SD 1.6) with an average duration of 3.3 min. Comparison with local bathymetry indicated that the majority of dives were to the seabed, signifying benthic foraging (Fig. 1c).

**Table 1:** Summary of tagged individuals performing multi-day foraging trips. PCAs are presumed prey capture attempts inferred from acceleration transients.

Animal I.D	Tagging date	No. offshore foraging trips	Recording length (days)	Length of foraging trip (days)	Sex (m/f)	Age class	Mean max dive depth (m) (SD)	Mean PCAs per day	Mean HVPs per day	Median distance made good (m) between PCAs (IQR)	Median distance travelled (m) between PCAs (IQR)
hs16_265a	21-Sept-2016	1	6.4	6	m	Adult	25 (12.7)	566	28	64.8 (95.5)	82.7 (112.1)
hs16_265b	21-Sept-2016	1^i^	23.2	> 5	f	Adult	19.6 (5.3)	821	19	42.6 (41)	62 (60.4)
hs16_265c	21-Sept-2016	1 2^ii^	21.7	8 > 8	f	Adult	18.6 (5.7) 17.5 (5.6)	756 845	18 14	46.2 (44.2) 40.4 (36.7)	63.9 (74.4) 59 (66.2)
hs17_109a	19-Apr-2017	1^i^	27.1	> 10	m	Adult	25.6 (9.2)	556	115	46 (61.6)	73 (78.3)
hs17_109b	19-Apr-2017	1^i^	30.6	> 5	m	Adult	33.1 (12.6)	390	10	45.7 (63.5)	80 (87.8)
hs17_109e	19-Apr-2017	1 2	26.8	7 4	m	Adult	20.3 (11.1 18.4 (5.1)	582 508	23 14	48.9 (58.6) 50.2 (66.8)	70.4 (77) 75.3 (86.1)
hs17_283a	10-Oct-2017	1 2^ii^	25.2	9 > 3	f	Adult	22.4 (8.4) 19.8 (8.5)	628 728	17 28	39.3 (41.7) 38.4 (42.9)	66 (78.9) 66.3 (79.2)
hs17_283b	10-Oct-2017	1 2^ii^	21.6	10 > 4	f	Sub-adult	15.3 (3.5) 16.5 (4.8)	812 806	50 24	34.9 (36.9) 33.3 (35.8)	58.8 (54.4) 57.2 (54.9)

HVPs are ‘high value prey’ captures in which prey appear to be brought to the surface for handling. Distance made good is the straight-line distance between successive PCAs. Distance travelled is the total distance travelled by the animal between successive PCAs.

^i^ First foraging trip incomplete, ^ii^ second foraging trip incomplete.

### **Hypothesis 1**

Harbour seals travel offshore to access rich prey resources.

**Figure 2:**
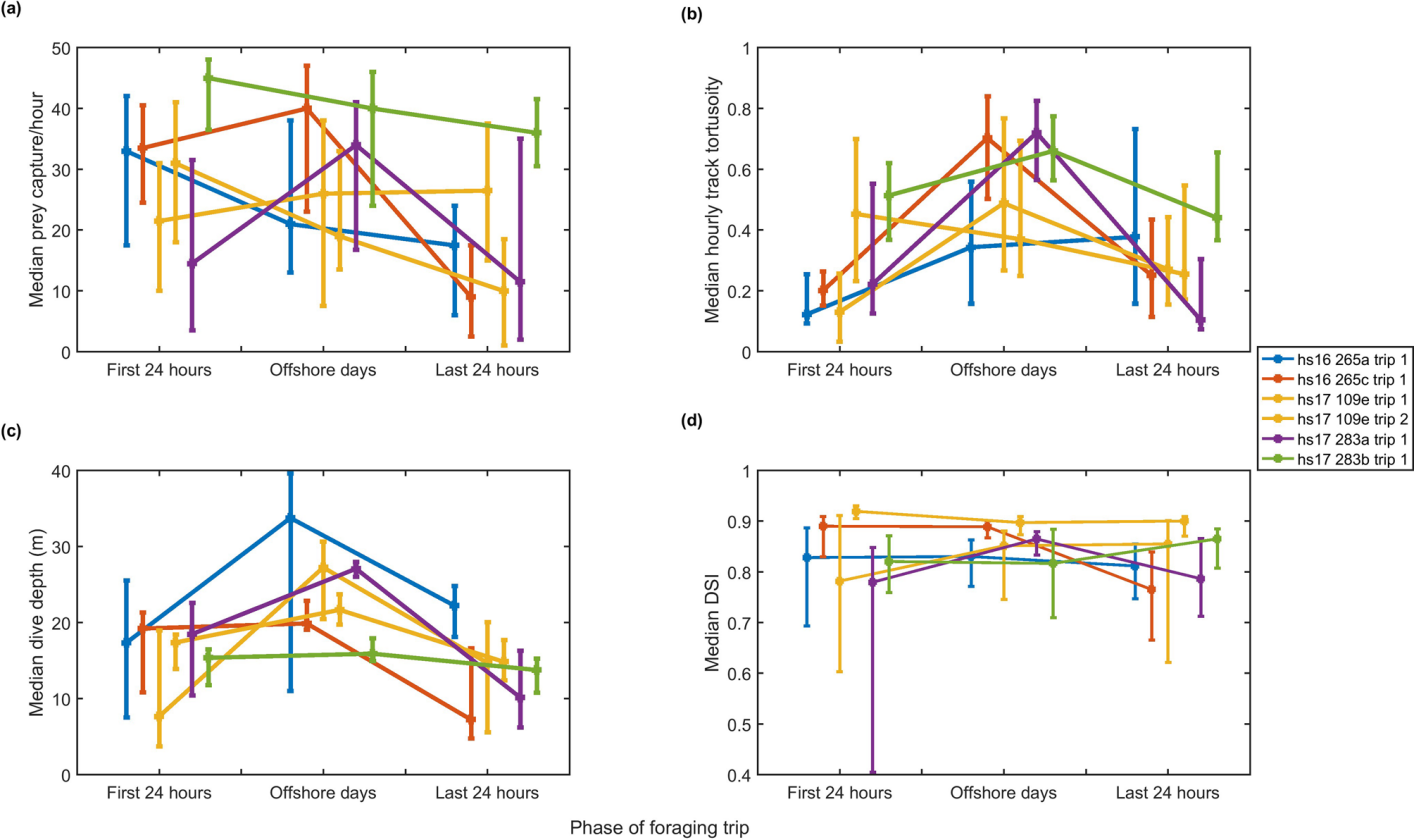
Median of (**a**) hourly PCA count, (**b**) hourly track tortuosity; (**c**) dive depth and (**d**) dive shape index (DSI) over the three phases of each foraging trip, with error bars representing upper and lower quartiles. ‘First 24 h’ encompasses the trip offshore from the haul-out. ‘Last 24 h’ encompasses the return trip to shore. ‘Offshore days’ encompasses all times in between. Colours represent individual foraging trips and six complete trips from five individuals are shown.

**Figure 3:**
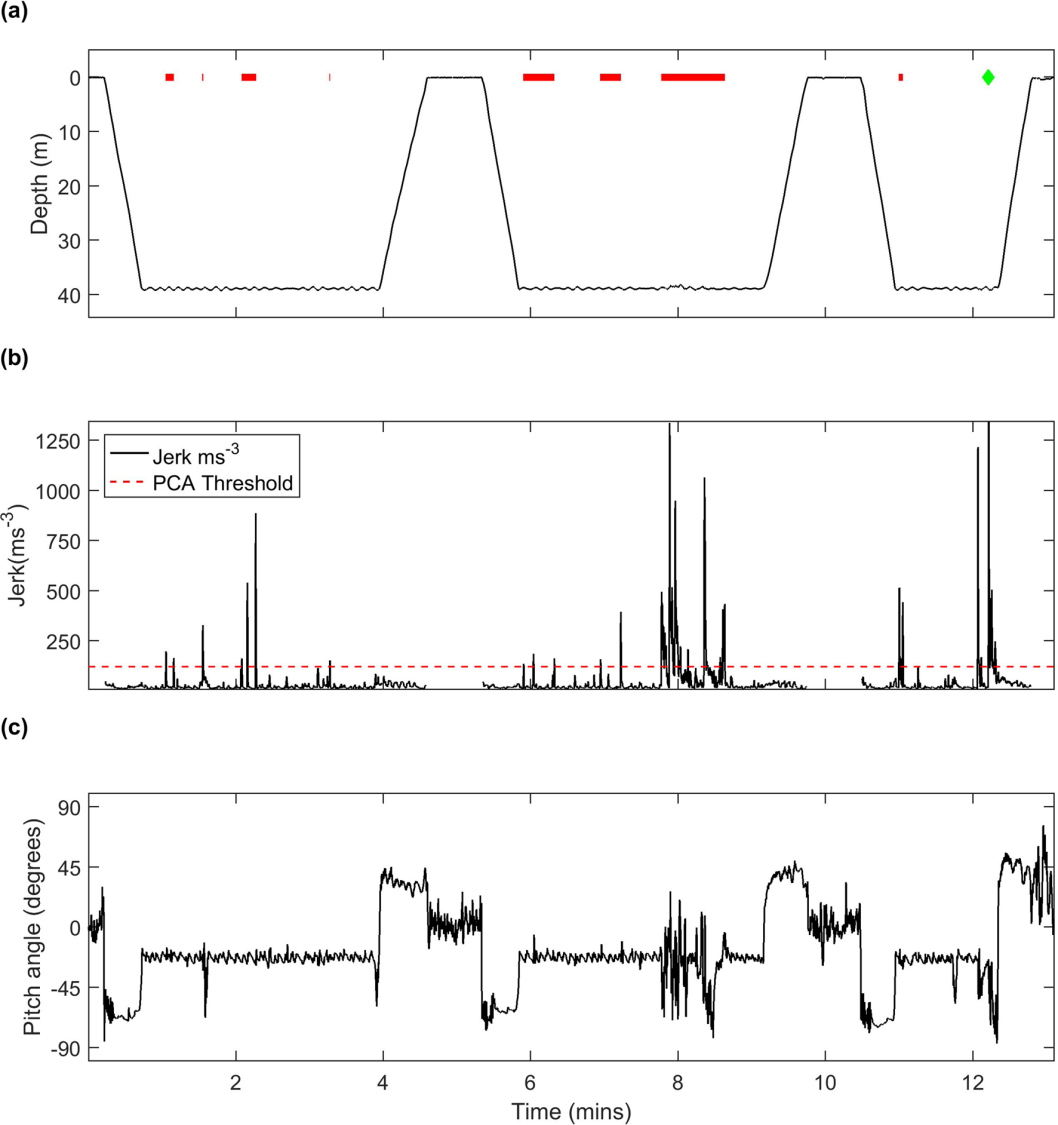
(**a**) Sequence of foraging dives from hs16_265a trip 1 showing the duration of detected prey capture events (PCAs) as red horizontal lines. The green diamond indicates a potential large prey capture (HVP) due to the jerk transient occurring near the end of an atypically short dive suggesting handling at the surface. (**b**) Root mean square of the norm jerk (200 Hz sampling rate, 0.4 s averaging time) over the same interval from which prey captures were inferred. The PCA detection threshold is indicated by the red dashed line. A blanking time of 20 s between PCA detections means that multiple jerks separated by less than this interval are interpreted as a single PCA, represented by individual horizontal red lines in (**a**). Gaps in jerk indicate where the signal has been blanked to prevent false PCA detections when the animal is at the surface. (**c**) Pitch angle of the animal over the same time interval. A negative pitch angle indicates a head-down posture and when this occurs at the base of dives benthic prey are likely being targeted.

As expected, the tracks of tagged seals showed intervals of largely straight-line travel as animals moved away from, and back to, the coast (Fig. 1a,b). Once offshore, the tracks became more varied with intervals of strong tortuosity (Figs. 1d and 2b). GPS outages occurred to the same extent during periods of travel as during offshore periods indicating that these do not explain differences in tortuosity within foraging trips (Supplementary Table S1). Despite the lower track tortuosity, median foraging rates inferred from jerk transients during 24-h intervals encompassing travel to and from offshore sites were within the range of median foraging rates during days spent offshore (Fig. 2a and Table 2). This suggests that the rate of foraging offshore is broadly comparable to the rate of foraging during travel to and from foraging sites and that harbour seals are continuously foraging even during what appears to be directed travel. This result is supported by harbour seals adopting a pitched-down body posture at the bottom of dives in all trip phases consistent with prey searching (Fig. 3). Prey capture attempts (PCAs) detected during the outward travel days alone were equivalent to 0.6–1.8 days of offshore foraging making the travel days significant contributors to the total intake in the trip. PCA detection rates are influenced by the blanking time chosen for the detector. This sets a trade-off between over-counting long interactions with single prey versus under-counting rapid series of short prey encounters. Using a reduced blanking time (10 s instead of 20 s) increased overall PCA rates but confirmed that foraging rates in travel days were comparable to those during days spent offshore (Supplementary Fig S1).

**Table 2:** Number of presumed prey captures (PCA) during each 24-h period of the foraging trip.

Foraging trip ID	First 24 h PCA count	No. of complete offshore days	Offshore days PCA count	Last 24 h PCA count
hs16_265a trip 1	703	4	881;696;453;318	391
hs16_265b trip 1	813	4	874; 841; 772;855	–
hs16_265c trip 1	749	6	855;827;737;890;801;827	342
hs16_265c trip 2	925	7	830;862;927;813;883;837;710	–
hs17_109a trip 1	918	9	909;631;512;392;387;438;438;406;558	–
hs17_109b trip 1	406	4	407;308;365;461	–
hs17_109e trip 1	533	5	760;753;599; 491;461	639
hs17_109e trip 2	702	2	583;427	271
hs17_283a trip 1	436	7	797;686;751;710;601;549;762	422
hs17_283a trip 2	594	2	677;791	–
hs17_283b trip 1	973	7	696;829;833;879;888;666;920	754
hs17_283b trip 2	732	2	813;863	–

The first and last 24 h encompass travel to and from an offshore site. For trips in which the tag recording ended early, the final 24 h is included in the offshore days.

**Figure 4:**
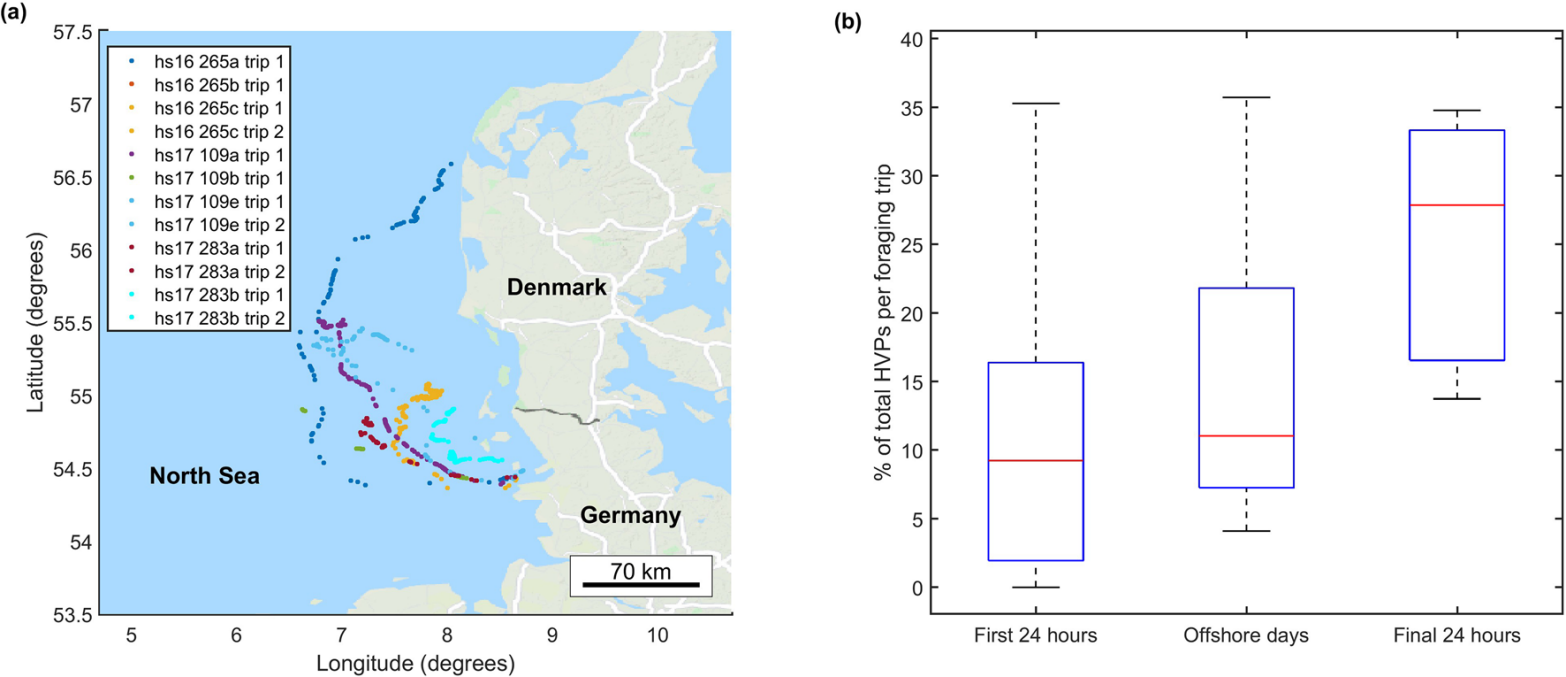
(**a**) Map showing the geographic location of potential high value prey captures (HVPs, i.e., a jerk transient occurring at the end of an atypically short dive). These events are not restricted to offshore sites but appear to occur across the foraging range. Map was generated in Matlab R2016a (http://www.mathworks.com/) using plot_google_map version 1.5 (https://au.mathworks.com/matlabcentral/fileexchange/27627-zoharby-plot-google-map). (**b**) % of total HVPs which occur at each stage of the foraging trip in 6 complete trips by 5 seals.

Short dives that ended in an acceleration transient suggesting capture and surface handling of high-value prey (HVP) were detected throughout foraging trips including travel days (Figs. 3 and 4a). Indeed, for most animals the highest proportion of HVPs occurred in the last days of the trip including the return journey (Fig. 4b). To control for potential bias in HVP counts due to shorter dives as animals approach shallower coastal waters during return travel, we repeated this test excluding dives shallower than 10 m and found a similar result. Typically, 15–25 large prey captures were detected per day but considerably higher rates were found in two offshore trips (hs17_109a trip 1: 115 HVPs/day and hs17_283b trip 1: 50 HVPs/day). While hs17_109a did not perform a subsequent foraging trip with which to aid comparison, hs17_283b did not have similar success in their next foraging trip indicating that perhaps larger prey have an unpredictable distribution or that seals do not target them consistently.

**Figure 5:**
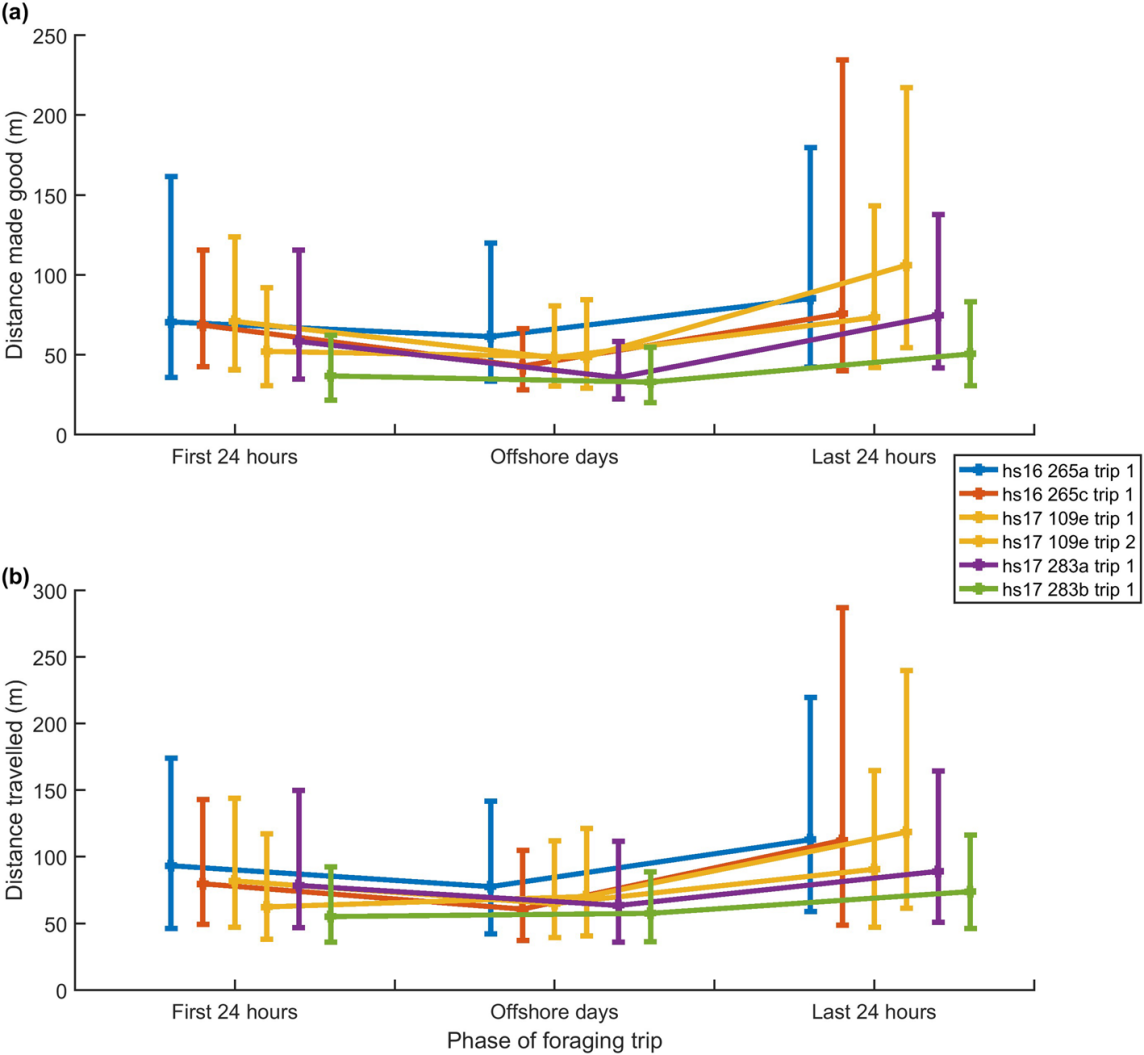
(**a**) Median distance made good between successive PCA locations over each phase of the foraging trip. (**b**) Median distance travelled between successive PCA locations over each phase of the foraging trip. Error bars in both plots represent lower and upper quartiles.

The median distance made good between PCAs (i.e., the straight-line distance between PCA locations) across all animals was 44.2 m. However, the inter-quartile range (IQR) for each animal was large, reflecting a high variability in prey distribution (Table 1). The median distance travelled between prey encounters (i.e., the sum of distances between samples in the dead-reckoned tracks) was 67.9 m also with a high IQR indicating variable search time for prey (Table 1). The median and IQR of these two inter-PCA distances were within the same range across all phases of the foraging trip for all animals (Fig. 5a,b) indicating that prey were not found in denser aggregations off-shore than during travel days.

### **Hypothesis 2**

Time spent resting increases over the course of an offshore foraging trip.

**Figure 6:**
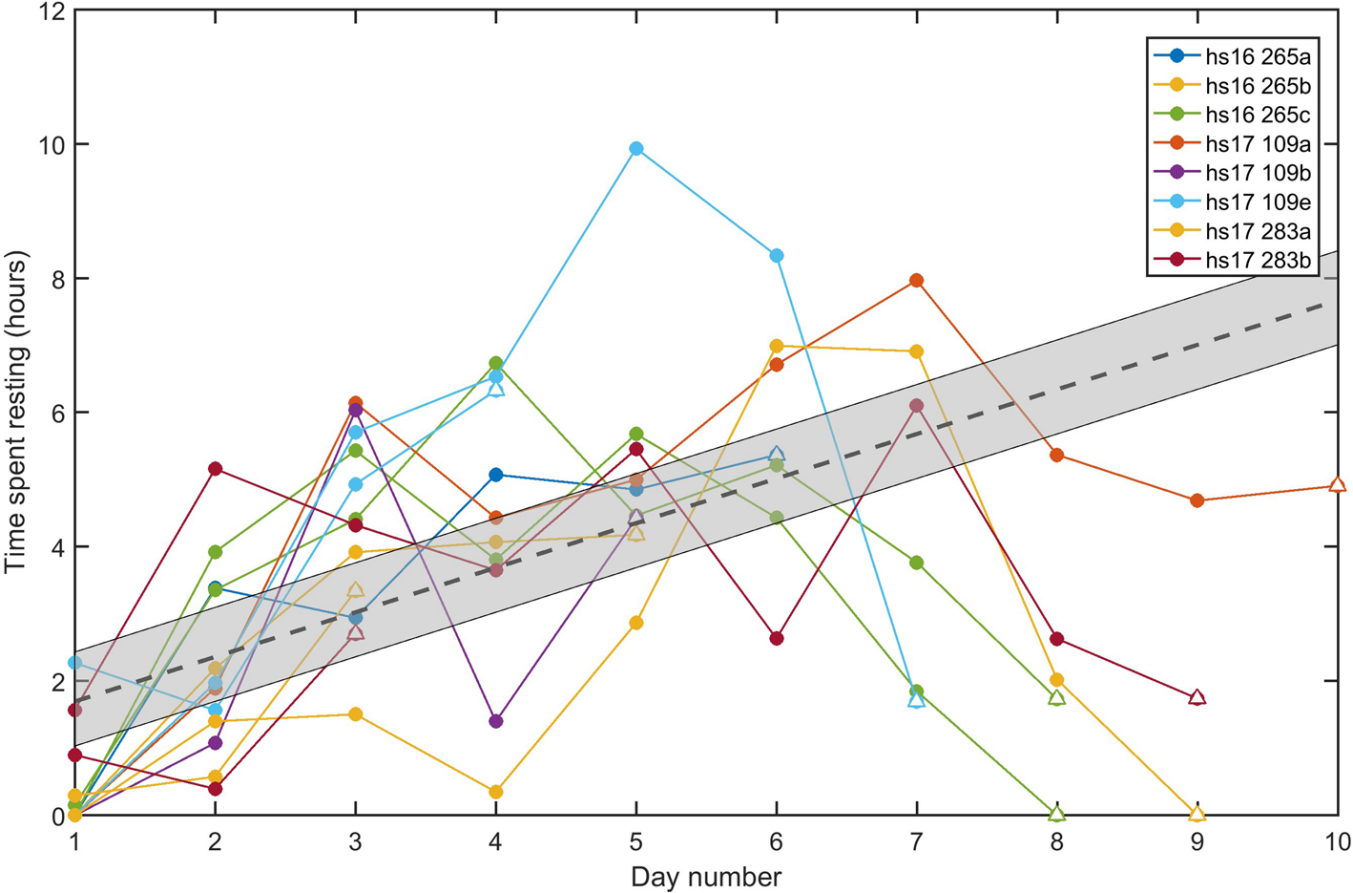
Rest time in hours during each complete 24-h period of the foraging trip starting when the seals leave the haul-out (incomplete 24-h periods at the end of the deployment are excluded). The dashed black line indicates the global mean model output, while the grey patch shows the range of model predictions for individual means across all animals. The return travel days, indicated with an open triangle, were not included in the regression as seals may postpone rest when they are returning to the haul-out.

Tagged seals rested for a median of 205.8 min/day, both at the surface and during distinctive low activity dives. Excluding the return travel day in which seals may postpone resting until they reach the haulout, a linear mixed effects model indicated a significant relationship between day number of the foraging trip and the length of time spent resting (*p* < 0.0001). The model fit showed an increase in resting time of 36.9 (SE 6.6) mins per day from the start of each foraging trip (Fig. 6) (marginal R^2^ = 0.38, conditional R^2^ = 0.46). Daily PCA count was not a significant covariate in the resting model (*p* = 0.06) indicating that foraging success may not be a substantial contributor to time dedicated to rest in a foraging trip.

Perhaps as a consequence of increased resting, daily PCA counts decreased by 24.8 (SE 6.5, *p* = 0.0003, marginal R^2^ = 0.08, conditional R^2^ = 0.62) for each day of the foraging trip. However, while the model was significant, the large difference between marginal and conditional R^2^ indicates that much of the variance in PCA rate stems from individual differences rather than linear changes with day number. While there was no significant relationship between day number and HVP count (*p* = 0.1), there was a trend for the lowest proportion of HVPs to occur on the outward journey and the highest proportion to occur on the return journey (Fig. 4b).

### **Hypothesis 3**

Track tortuosity and dive shape are robust indicators of foraging behaviour.

A linear mixed effects model showed a significant but weak relationship between PCA rate and tortuosity during offshore days when analysed across all time intervals (30 min interval: *p* = 0.0001, marginal R^2^ = 0.04, conditional R^2^ = 0.2; 15 min interval: *p* = 0.02, marginal R^2^ = 0.01, conditional R^2^ = 0.16; Dive interval: *p* = 0.002, marginal R^2^ = 0.01, conditional R^2^ = 0.16) suggesting that tortuosity is a weak predictor of foraging behaviour when animals are offshore.

In general, dive depth increased as animals went offshore and decreased as they returned to the coast (Fig. 2c), consistent with a gently sloping seabed away from the coast. The dive shape index however stayed relatively constant and high throughout all phases of the foraging trip (Fig. 2d), consistent with continuous benthic foraging. U-shaped dives occurred both when animals were foraging and resting making it difficult to differentiate these behaviours with dive data alone, but as resting dives comprised only 7.5% (SD 3.6) of total dives their impact on the hourly mean dive shape index was minimal. Thus, the prevalence of U-shaped dives in our data supports a high time investment in foraging but has little value for predicting PCA rates.

## Discussion

Our understanding of harbour seal foraging behaviour has largely been drawn from inferences based on surface movement patterns and dive shape acquired at low sampling rates from biotelemetry tags^23^. Although fine-scale movement sensors (accelerometers and magnetometers) have previously been used on harbour seals in the German Wadden Sea^8^, sampling rates were too low (maximum of 1 Hz) to detect the fast movements associated with prey capture attempts^37^. Here we combine high-resolution bio-logging data from GPS, depth, accelerometer and magnetometer sensors to examine fine-scale harbour seal foraging and resting behaviour during multi-day offshore foraging trips. In other pinniped species, offshore provisioning trips are performed to target more abundant or higher quality resources^2–5,41^. However, using distinctive acceleration transients as a proxy for prey capture attempts (PCA)^37^, we found that harbour seals forage almost continuously throughout the trip including on the outbound and return journeys, suggesting that offshore patches are not so rich as to make opportunistic foraging during travel inefficient.

### Indicators of foraging behaviour

Our interpretations are dependent on the assumption that PCAs can be identified from acceleration transients and, as with any proxy of behaviour, this metric is prone to error. Prey strikes and handling involve rapid motion of relatively small cranial muscles, leading us to choose a high acceleration sampling rate (200 Hz) along with a processing method (the RMS jerk) that emphasises high frequency components^37^. This resulted in discrete high magnitude peaks in jerk during presumed foraging dives that are clearly distinguishable from the much lower jerk levels during active swimming (Fig. 3b), reducing the possibility of confounding prey interactions with other activities. Nonetheless, the choice of detection threshold and blanking time will inevitably lead to under-counting of PCAs that are close together or that require little head movement. However, the primary goal of this study was to identify relative foraging rates as a function of location and so, for our interpretations to be incorrect, there would need to be a strong correlation between prey attack behaviour and location. The uniformity of foraging posture (i.e., pitched down to accommodate searching along the bottom) throughout our data (e.g., Fig. 3) suggests that there should be little spatial variation in the detectability of PCAs. We therefore argue that jerk transients offer a consistent indication of where and when harbour seals encounter prey, independent of larger-scale movement patterns, as has been found for other marine mammal species^31,33,34^.

### Foraging occurs throughout offshore trips

Assessments of where harbour seals forage have largely come from low resolution position and dive data^10,19,42^. An underlying assumption is that foraging occurs primarily during area restricted movements at the surface, and that straight-line movements must therefore represent travelling^19,42^. Whilst the possibility of opportunistic feeding during travel has been considered^23,42^, the contribution to total intake has been assumed small leaving the notion that offshore feeding trips serve to access a distant valuable food resource. Using acceleration transients to infer foraging reveals a different picture. Animals in our study encountered prey during both tortuous and straight track segments, giving only a weak association between tortuosity and the rate of PCAs at all timescales tested (Figs. 1d and 2). This suggests that prey were encountered, not in ‘hot spots’ but sporadically across the entire foraging range. Thus, the conventional decomposition of low resolution movement data into mutually exclusive travel and foraging episodes based purely on straightness of track may be overly simplistic for harbour seals at this site. Dive shape appears to be a similarly imprecise predictor of foraging rates in our data because of near-continuous foraging and low variety in dive shape (Fig. 2d). Moreover, the one behavioural state in which foraging does not occur, resting, involves dive shapes which can easily be confused with foraging in low-resolution depth data.

Our results suggest that whilst harbour seals intend to travel offshore directly, as evidenced by relatively straight-line movement, this is done in a way that maximises the opportunity for foraging along the way. The majority of dives during the outward and return trips are benthic and seals adopt the same pitch-down posture at the base of these dives that they use during offshore foraging dives, consistent with searching for benthic prey (Fig. 3). This posture and depth are less than optimal for travel so foraging along the way appears to be important enough to sacrifice some travel efficiency, suggesting in turn that offshore prey patches are not so dense as to make opportunism inefficient while underway. Thus, continual patch assessment may be an important strategy in multi-day foraging trips rather than a reliance on predictable rich offshore locations.

### Why do harbour seals travel so far offshore to find food?

If prey are available throughout their foraging range, why do animals endure the extra costs of travelling further from haul-outs? One suggestion is that prey captured offshore are larger. The shallow waters of the Wadden Sea provide nursery grounds for the juveniles of many fish species in spring and summer with most of these migrating into deeper offshore waters in autumn and winter^15,16^. To test if seals took larger prey offshore, we detected short dives that ended with a PCA interpreting these as indicative of a high-value prey (HVP) being brought to the surface. These detections likely underestimate the number of HVPs taken but provide a relative indication of where and how often such prey is encountered.

Although HVPs were found throughout foraging trips, there was a trend for the lowest proportion of HVPs to occur on the outward journey and the highest proportion of HVPs to occur on the return journey with many individuals having the largest daily count in the final 24 h when they are returning to the haul-out (Fig. 4b). Shorter dives in the shallow waters near the coast may lead to an over-estimation of HVP detections in the travel days but a similar result was found when these dives were excluded. It therefore appears that larger prey are available across the foraging range but that less time is dedicated to their pursuit during outward trips, possibly because the handling time make them a less efficient food source when balancing travelling to more distant food resources with foraging along the way. Taken together, these results lead us to conclude that harbour seals do not appear to be encountering more or larger prey further offshore and therefore cannot explain why they travel as far from the coast as they do.

For most individuals the mean rate of HVPs was 15–25/day (compared to ~ 600 PCAs/day), and so these may not represent a significant portion of the diet. However, for two individuals encounter rates of large prey were much higher (50–115/day) but inconsistent between consecutive foraging trips, suggesting that if there are individual tactics to target these prey, they are not uniformly successful.

If prey are distributed relatively uniformly, this does not necessarily make it optimal to forage close to the haul-out. Whilst harbour seals congregate in large numbers at haul-out sites, they are solitary hunters^43^. Prey resources closer to the haul-out will be depleted quickly due to conspecific competition in an Ashmole’s halo phenomenon that has been reported for other colonial species^18,44,45^ . For harbour seals, access to nearby resources may be controlled by dominant animals while subordinate individuals may choose to forage further from the haul-out to avoid competition. However, larger individuals that are better able to afford the transport costs of travelling further offshore would also be rewarded with patches that are less exploited by conspecifics. Indeed, some studies show for both harbour and grey seals that the largest individuals travel the farthest and spend the longest time away from the haul-out^7,46^. Indeed, these larger individuals tend to be males leading to sexual segregation in foraging areas^8^. However, studies in other locations have shown opposite results^47^ suggesting that resource partitioning may be site specific. We found no clear pattern in our own data to support either of these suggestions. In either case, our data does not provide evidence for a single rich offshore foraging zone. Therefore, we posit that harbour seals performed multi-day foraging trips to access widely distributed prey and to reduce competition with conspecifics.

### The role of rest in deciding to return to the haul-out

Our results show that resting time at sea increases over the course of a foraging trip by some 37 min per day, possibly indicating an accumulating need for rest (Fig. 6). Increased resting comes at the expense of foraging time and we found a daily decrease of about 25 PCAs. Over a 5-day foraging trip this equates to ~ 17% reduction in intake between the first and final days, assuming an average first day PCA rate of ~ 700 and that prey size and quality remain constant. Whilst resource depletion also reduces foraging efficiency, this is less likely to be a factor due to the large area covered during foraging trips.

Rest may serve several functions including sleep, digestion, lactate processing and vigilance. Brasseur et al.^48^ showed that when prevented from hauling out, harbour seals compensated by later increasing time spent hauled out suggesting that resting in the water is not equivalent to hauling out. Other pinniped species show uni-hemispheric sleep while in the water with an almost complete absence of R.E.M sleep^49^. Therefore, harbour seals may compensate for a lesser quality of rest at sea by devoting more time to it throughout a foraging trip, until it eventually becomes more economical to return to the haul-out. Thus, although other factors likely influence the length of foraging trips, we suggest that loss of foraging time due to increased time spent resting could be a major driver in the decision to end a multi-day foraging trip.

### Perspectives

Taken together, we show that the harbour seals assessed in this study did not rely on specific rich offshore foraging zones. Instead, animals appear to travel offshore to access broadly distributed prey and potentially avoid intra-specific competition. Our study includes sub-adult and adult animals of both sexes and covers two seasons (spring and autumn) but the relatively small sample size precludes the exploration of temporal variability or whether specific life history events such as breeding and pupping influence foraging behaviour. While previous studies have looked at the effect of sex on space utilisation by harbour seals in the Wadden Sea^8^, they have not looked at how life history and age influence fine-scale behaviour. This should be a consideration in future fine-scale behavioural studies, in order to identify key foraging areas associated with life cycle stages e.g., pregnant females and juveniles.

The harbour seal population in the German Wadden Sea has expanded considerably since a ban on hunting in the mid-1970s as well as the establishment of national parks^43^, despite two outbreaks of phocine distemper virus^50^ and one die-off due to Influenza A virus^51^. But even though harbour seals in the Wadden Sea appear to be doing well^52^, it is pressing to understand how planned expansion of offshore renewables could impact these animals. Given the broadly distributed resources exploited by animals in our study, harbour seals may be relatively resilient to disturbances at fixed locations such as offshore construction sites. However, our results also indicate that seals feed nearly continuously during offshore trips and therefore may be vulnerable to cumulative effects of brief disturbances such as ship passes^22^ or of intense noise sources that have large zones of impact^53^. Moreover, the re-colonisation of grey seals in the area^54^ may add further resource competition. These factors are compounded by intensive fishing in the North Sea since the 1960s, leading to changes in the structure of food webs^55^ as well as providing direct competition for resources. Studies have hinted at decreases in the size of North Sea fish over the last half century^56^ with further decreases projected due to the effects of warming seas^57^. These pressures likely apply to harbour seals across many locations raising the importance of collecting comparable data in other study sites and populations. We argue that bio-logging tags that collect high-resolution data from a greater range of movement sensors offer important advantages when interpreting the behaviour and foraging performance of harbour seals. Such detailed behavioural data could potentially lead to more informed and effective conservation measures for this species.

## Materials and methods

### Fieldwork

Ten seals were tagged in September 2016, and in April and October 2017. Adult and sub-adult harbour seals (Table 1) were captured at low tide adjacent to haul out sites at Lorenzensplate (54.44°N, 8.64°E), Germany. Capture and handling were carried out in accordance with relevant guidelines and regulations and followed the methods in Hasselmeier et al.^58^. The study was approved under animal experiment ethical permit number Az V312- 72241.121-19 (70-6/07) and V244-3986/2017 (17-3/14) of the Ministry of Energy, Agriculture, Environment and Rural Areas of Schleswig–Holstein, Germany.

Each animal was instrumented with a DTAG-4 joined with an Argos transmitter (SPOT-6), floatation and timed release with a combined weight of 206 g (see Mikkelsen et al. for details^22^). The DTAG-4 is an archival tag containing synchronously sampled sensors for sound (not used here), motion and position, with a recording duration of up to 30 days. The depth sensor and tri-axial magnetometer were sampled at 50 Hz while the tri-axial accelerometer was sampled at 200 Hz. A snapshot GPS^59^ acquired up to three positions every 3 min when the animal was at the surface.

### Data processing

Processing was carried out using custom tools (www.animaltags.org) in Matlab R2016a (The Mathworks, Natick, MA, USA). Data were divided into individual offshore foraging trips (i.e., intervals of > 24 h without hauling out) extending from departure to the return to haul-out (or the end of the recording if that occurred first). Tagged seals also performed short inshore foraging trips between regular daily haul-outs but these data were excluded from our analyses as previous work suggested that most foraging in this area occurred on multi-day trips^10,11^.

Pressure, accelerometer and magnetometer data were decimated to 5 Hz to calculate dive profile, posture and dead-reckoned tracks within each foraging trip. Dives deeper than 2 m were analysed for dive shape and bottom time. A dive shape index was computed by dividing the sum of all depths sampled within the dive by the product of the maximum depth and the total number of depth samples (i.e. giving values between 0 and 1, with values closer to 1 indicating a more U-shaped profile). The bottom time of each dive was defined as the time spent within 70% of the maximum depth. Posture, parameterised by pitch, roll and heading angles, was estimated following Johnson and Tyack^60^.

The conventional dead-reckoning procedure which assumes that animals move in the direction of their longitudinal axis^8,40^ was not appropriate here as seals frequently moved horizontally at the base of dives (as inferred from dive depth) but with a downwards pitched posture consistent with searching for prey on the seafloor. To avoid errors due to the mismatch between pitch angle and movement direction, we adopted a 2-dimensional dead-reckoning method in which the horizontal track was calculated by integrating the heading vector of the animal (i.e., [cos(h), sin(h)] in a North-East frame, where h is the true heading) multiplied by an estimated horizontal speed. As the tag does not include a speed sensor, we used a horizontal speed estimate of $\sqrt {1.4^{2} - depthrate^{2} }$ where 1.4 m/s is the typical forward speed for harbour seals^8^, and depth rate, constrained to a maximum value of 1.4 m/s, is computed from the differential of the depth data. The dead-reckoned track was then corrected to match GPS positions at the surface by adding a constant vector to each track point between pairs of GPS positions, effectively correcting for swim speed, heading errors and water currents.

To detect potential prey capture attempts (PCA), the norm jerk^37^ (i.e., the vector magnitude of the acceleration differential) was calculated from 200 Hz accelerometer data. The root-mean-squared (RMS) of the norm jerk was then taken over 0.4 s intervals with 50% overlap to give an RMS jerk measure with a sampling rate of 5 Hz. A peak detector was used to locate transients in the RMS jerk that may indicate PCAs, requiring selection of a blanking time and detection threshold. The blanking time specifies the minimum time between detections to avoid making multiple detections during prolonged prey capture and handling. The histogram of time between jerk peaks for our data did not indicate a simple two-process model^61^ from which a blanking time could be inferred. Harbour seals forage on a range of prey sizes and while small fish can be captured within a few seconds, larger prey such as flat fish require longer handling. We accordingly used pursuit, capture and handling times of harbour seals feeding on sand eels and flounder^62^, weighted by the approximate relative occurrence of these in the diet to calculate a blanking time of 20 s. Using a fixed blanking time risks under-counting sequential aggregated small prey while over-counting prey that require extensive handling, and so will likely not give accurate absolute PCA counts. However, this method is suitable for assessing relative prey encounter rates across foraging trips provided that the proportion of prey types does not change greatly with time. To ensure the robustness of our results to this assumption analyses were repeated with a shorter blanking time of 10 s.

As the magnitude of RMS jerk is affected by tag placement as well as potentially by animal size^63^, a detection threshold was chosen for each seal based on the RMS jerk during dive descents and ascents when seals are less likely to be chasing prey. A gamma distribution was fit to the squared RMS jerk during these periods and the square-root of the value corresponding to a cumulative probability of 0.9999 was used as the threshold above which RMS jerk peaks were considered prey capture attempts. This threshold implies a false detection on average every 33 min of active swimming (i.e., 1 false alarm per 10,000 samples at the data rate of 5 Hz) which is much lower than the typical prey encounter rate. Jerk transients can arise for a number of reasons unrelated to foraging: wave action at the surface, collisions of the tag with the seabed and possibly as a startle response to disturbance. Harbour seals typically target benthic and mid-water prey and so to reduce detection of splashes at the surface, peak detection was disabled at depths of < 1 m. Given the dorsal location of the tag on the body, contact with the seafloor requires that the animal is upside down. Such postures occurred rarely in foraging dives but were frequent during resting. To eliminate false detections due to seafloor contact, jerk peaks detected when the accelerometer ventro-dorsal axis was negative were ignored.

Harbour seals are known to bring large prey to the surface to facilitate handling^64^ whereas smaller prey are ingested on-the-go during dives. Examination of our data showed that while most foraging dives were longer than 150 s and contained several PCAs, a subset of dives to similar depths were notably shorter and contained a strong jerk transient just prior to the ascent. We interpreted these short dives (i.e., with duration < 150 s and a PCA within 20 s of the end of the bottom time) as indicative of high-value prey (HVP) captures that required surface handling. This method will under-estimate large prey captures, as some will occur at the end of dives with typical duration. However, this bias should be independent of foraging trip phase making it a useful relative measure.

The instantaneous stroking rate, a proxy for swimming effort, was calculated by detecting individual strokes in the magnetometer data^65^. The dominant stroking frequency (DSF, sensu Sato et al.^66^) for each animal was first calculated and the magnetometer lateral axis data were then high-pass filtered with a cut off frequency of 0.5 DSF for each animal to remove low frequency postural changes^35^. A hysteretic detector with a 3 μT threshold was used to detect swim-strokes in the filtered signal.

### Behavioural states

Although a previous study identified rest, travel and foraging states in at-sea data from harbour seals^21^, we found few dives with active swimming that seemed solely for travel. In comparison, resting was readily distinguishable from active behaviours (i.e., foraging/travel) and data were therefore divided into two ethographic states. Periods of rest were identified from dive and stroking rate data and comprised either extended intervals at the surface or sequences of dives with slow descents^21^ and exceptionally low stroking rates at the bottom. Resting dives often also contained distinctive slow rolling movements at the base of the dive^22^.

### Data analysis

To compare times spent mostly travelling to and from offshore sites versus time spent offshore, foraging trips were divided into two travel phases (i.e., the first and last 24-h period of each trip) and an offshore phase comprising the remainder of the data. The predominantly straight-line movements away from/to shore tended to last about 24 h for all animals but this interval was chosen primarily from the need to average over diel changes in prey and predator behaviour. For animals with incomplete foraging trips (i.e., the recording ended before the animal reached the haul-out), the final 24 h were subsumed in the offshore phase. To quantify changes in resting behaviour during offshore trips we used a linear mixed effects model using the R (R Development Core Team, 2019) package nlme, to predict hours spent resting per day as a function of trip day number and number of daily PCAs. The return travel days (i.e., the final 24 h) were not included in the regression as seals may postpone rest when they are returning to the haul-out*.* Trip number (1 or 2) was nested in animal ID (1-8) as random intercepts. The model was validated using tests for normally distributed residuals, homoscedasticity and serial correlation. Goodness of fit was assessed using conditional and marginal R^2^ values, computed using the R package MuMIn^67^. Marginal R^2^ evaluates the variance in the data explained by the fixed effects alone, while conditional R^2^ evaluates the variance explained by both fixed and random effects.

To compare foraging inferences from our high-bandwidth acceleration data to those taken from movement patterns and dive shapes, we computed hourly median and max dive depth values as well as hourly median and max dive shape indices across the three phases of each offshore trip. Track tortuosity was also compared over the three phases. Tortuosity was calculated on an hourly basis as: (distance travelled − distance made good)/distance travelled, where distance made good is the straight-line distance between the first and last track point of each hour and distance travelled is the stretched-out track travelled by the seal. Gaps in GPS data can occur when the tag does not clear the surface for long enough or when the sea-state is high. To assess the potential impact of these outages on tortuosity during each phase of the foraging trip, median time differences between GPS outages were evaluated at each phase.

To evaluate in more detail the use of track tortuosity as an indicator of foraging behaviour during the offshore phases of trips, tortuosity was calculated over 3 timescales: 30 min, 15 min and over individual dives. The resulting tortuosity values, as well as the PCA counts over the same time intervals, were subsampled to reduce serial correlation (every 6th 30 min interval, every 10th 15 min interval, and every 20th dive were used). Linear mixed effects models were then constructed in R to predict track tortuosity from PCA count with a separate model for each timescale. Trip number (1 or 2) was nested in animal ID (1-8) as random intercepts in each model and models were validated as described previously.

## Supplementary Information

Supplementary Information

## Data Availability

Data is available at 10.6084/m9.figshare.14171315.
