# Supplementary material for: Drivers and constraints on offshore foraging in harbour seals
Source: Sci Rep. 2021 Mar 22;11:6514. doi: 10.1038/s41598-021-85376-2 (PMC7985366; doi:10.1038/s41598-021-85376-2)
Supplement: Supplementary file 1 — Supplementary Information [file 41598_2021_85376_MOESM1_ESM.docx]

**Drivers and constraints on offshore foraging in harbour seals.**

**Vance, H.M^1*^, Hooker, S.K^1^, Mikkelsen, L^3^, van Neer, A^2^, Teilmann, J^3^, Siebert, U^2^ & Johnson, M^,4^.**

*^1^ SMRU (Sea Mammal Research Unit), University of St Andrews, St Andrews, Fife KY16 8LB, UK. ^2^ Institute for Terrestrial and Aquatic Wildlife Research, University of Veterinary Medicine Hannover, Foundation, Werftstraße 6, 25761 Büsum, Germany. ^3^ Marine Mammal Research, Department of Bioscience, Aarhus University, Roskilde, Denmark. ^4^ Department of Biology, Aarhus University, Aarhus C, Denmark.*

*Email* [–hmv@st-andrews.ac.uk](mailto:–hmv@st-andrews.ac.uk)

**Supporting Information**

*Table S1. Median, 75^th^ percentile and max time differences (minutes) between successive GPS positions during 3 phases of offshore foraging trips. Maximum GPS outages did not differ appreciable between travel and offshore days.*

| *Foraging Trip I.D* | *First 24 hours* | | | *Offshore days* | | | *Last 24 hours* | | |
| --- | --- | --- | --- | --- | --- | --- | --- | --- | --- |
|  | *Median* | *75%ile* | *Max* | *Median* | *75%ile* | *Max* | *Median* | *75%ile* | *Max* |
| *hs16_265a trip 1* | *4.72* | *5.54* | *165.97* | *4.28* | *6.62* | *465.52* | *3.72* | *8.62* | *196.51* |
| *hs16_265b trip 1* | *3.78* | *4.51* | *491.72* | *4.46* | *6.45* | *395.45* | *-* | *-* | *-* |
| *hs16_265c trip 1* | *4.82* | *5.65* | *53.01* | *4.06* | *4.57* | *198.41* | *3.43* | *4.08* | *13.74* |
| *hs16_265c trip 2* | *4.56* | *4.94* | *27.01* | *4.05* | *4.65* | *189.75* | *-* | *-* | *-* |
| *hs17_109a trip 1* | *3.71* | *4.13* | *117.64* | *4.01* | *8.39* | *712.73* | *-* | *-* | *-* |
| *hs17_109b trip 1* | *5.15* | *6.59* | *224.39* | *5.44* | *9.25* | *349.05* | *-* | *-* | *-* |
| *hs17_109e trip 1* | *5.07* | *15.50* | *303.45* | *8.47* | *19.46* | *788.96* | *7.22* | *12.94* | *295.67* |
| *hs17_109e trip 2* | *5.40* | *10.95* | *429.38* | *4.88* | *12.32* | *245.89* | *4.25* | *11.95* | *167.62* |
| *hs17_283a trip 1* | *3.81* | *4.63* | *78.82* | *3.66* | *4.09* | *117.65* | *3.61* | *4.50* | *24.49* |
| *hs17_283a trip 2* | *4.28* | *4.96* | *47.69* | *3.81* | *4.17* | *29.54* | *-* | *-* | *-* |
| *hs17_283b trip 1* | *3.63* | *4.46* | *70.46* | *4.23* | *7.64* | *390.74* | *3.16* | *4.23* | *71.47* |
| *hs17_283b* *trip 2* | *3.96* | *4.86* | *102.66* | *3.65* | *4.89* | *232.00* | *-* | *-* | *-* |


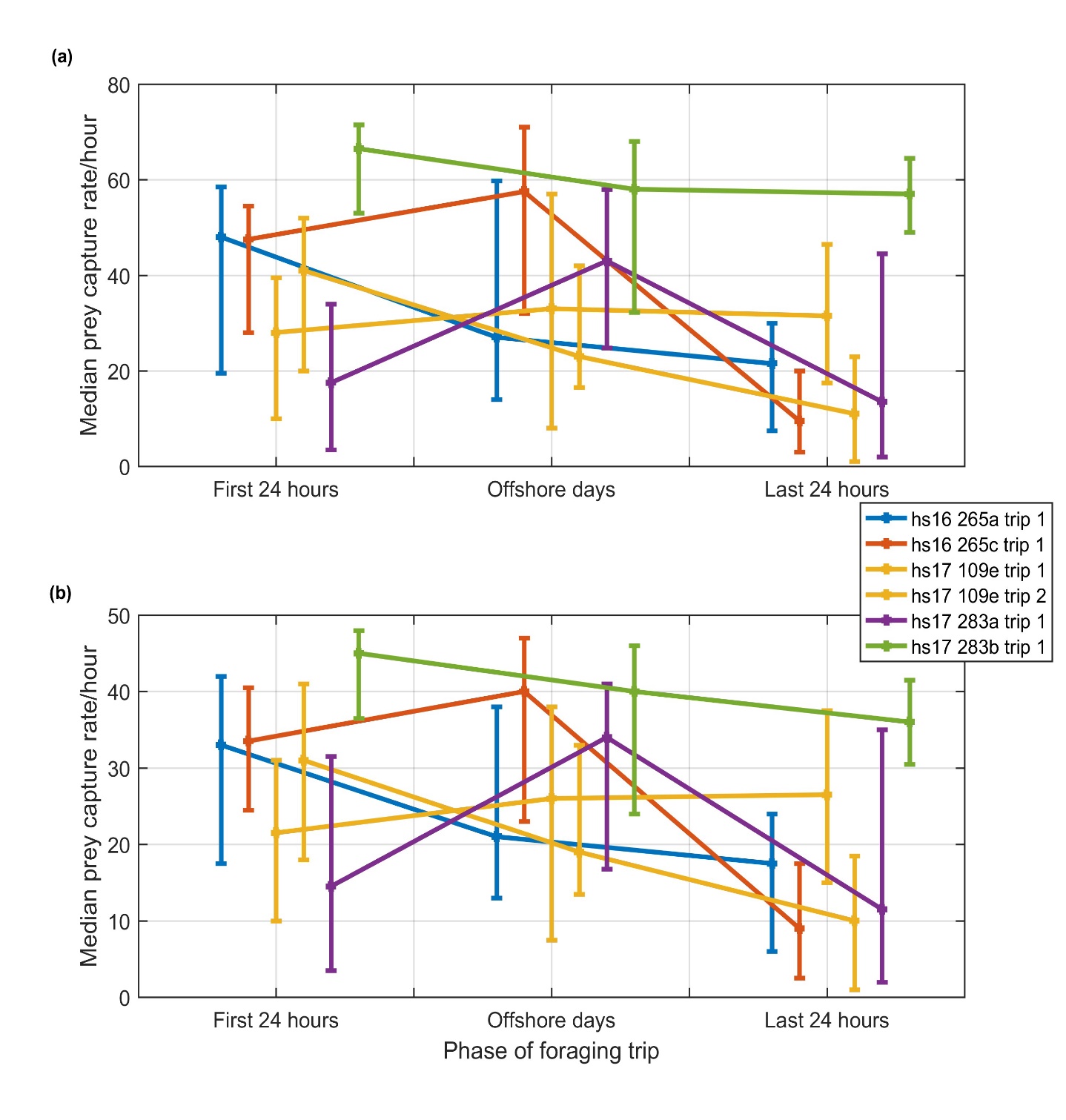


*Fig. S1. Median of hourly PCA count, with (a) a 10 second blanking time and (b) a 20 second blanking time between subsequent PCAs and over the three phases of the foraging trip, with error bars representing upper and lower quartiles. Whilst the absolute number of PCAs changes by using different blanking times the relative number of PCAs across the different phases of the foraging trip is relatively similar.*
